# Determinants of trust among ethnic minority community members towards community-based organisations and leaders during pandemics and other emergencies in Australia

**DOI:** 10.1136/bmjgh-2025-019445

**Published:** 2026-08-03

**Authors:** Holly Seale, Pippa McDermid, Danielle H Heinrichs, Helen Bromhead, Katrina Blazek, Michael Camit, Nadia Chaves, Mitchell Smith, Cliff Goddard, Abdullah Masud, Lisa Woodland, Jim Macnamara, Mashreka Sarwar, Harris-Roxas Ben

**Affiliations:** 1 School of Population Health, University of New South Wales, Sydney, New South Wales, Australia; 2 Institute for Educational Research, Griffith University Griffith, Mount Gravatt, Queensland, Australia; 3 School of Humanities, Languages and Social Science, Griffith University, Nathan, Queensland, Australia; 4 Multicultural Services, South Western Sydney Local Health District, Liverpool, New South Wales, Australia; 5 Alfred Health, Melbourne, Victoria, Australia; 6 NSW Refugee Health Service, Sydney, New South Wales, Australia; 7 South Eastern Sydney Local Health District, Kogarah, New South Wales, Australia; 8 School of Communication, University of Technology Sydney, Sydney, New South Wales, Australia; 9 Institute of Health Innovation, Macquarie University Australian, Sydney, New South Wales, Australia

**Keywords:** Public Health, Health policy, Global Health, COVID-19, Health education and promotion

## Abstract

**Background:**

Effective pandemic plans must be applicable to all members of our communities. However, people from multicultural communities with diverse language needs often engage with a wide range of organisations and individuals when seeking information during a pandemic or health emergency. Despite this, there is limited evidence on levels of trust and the influence of these sources on promoting community engagement with pandemic preparedness activities in high-income settings.

**Methods:**

A cross-sectional survey was conducted among members of ethnic minority communities in Australia who self-identified with one or more of six selected language or cultural groups. The survey, available in translated online and paper formats, incorporated several validated instruments and included both closed and open-ended items. Cross-tabulation analyses were used to examine associations between participant characteristics and levels of trust in community organisations and community leaders during the COVID-19 pandemic.

**Results:**

590 completed responses were included in the study. Participants were aged between 18 and ≥95 years old and identified as: Arabic (28.7%), Chinese (24.7%), Bangladeshi (22.3%), Pasifika (10%), Nepalese (8.2%) or Ezidi (6.1%). Predictors of moderate-to-very high trust in community-based organisations (CBOs) included participants who had arrived in Australia ≥20 years ago and had previously sought information from community groups, local councils and community workers. Likewise, moderate-to-very high trust in community leaders was more common among those who were more willing to seek assistance, arrived in Australia ≥20 years ago, were aged between 35 and 44 and had higher trust in community groups.

**Conclusions:**

Results indicated that trust in CBOs and community leaders was associated with willingness to seek assistance, satisfaction with communication efforts, trusted local councils and longer settlement in Australia; however, these associations may not reflect the reach or influence of community organisations and leaders across all ethnic minority groups.

**WHAT IS ALREADY KNOWN ON THIS TOPIC**

- Ethnic minority populations are disproportionately affected by crises and disasters globally, partly due to limited access to clear and accessible information. Understanding the factors that influence trust in sources of vital health information during emergencies is critical to supporting effective communication with ethnic minority communities during a health crisis.

**WHAT THIS STUDY ADDS**

- Levels of trust in community leaders and community-based organisations differed across community groups and were positively associated with individuals’ likelihood of seeking assistance or information.

**HOW THIS STUDY MIGHT AFFECT RESEARCH, PRACTICE OR POLICY**

- Government leaders, policymakers and community-based organisations need to examine the sources of information and trust in communication sources of ethnic minority populations when addressing crisis communication and pandemic planning.

## Background

 To encourage participation in pandemic containment activities or other emergency response actions, such as those to reduce the health impacts of heatwaves (eg, drinking water, dressing lightly, seeking cooler settings such as shopping centres/religious venues), communities need to be effectively engaged.^1 2^ This is particularly important for ethnic minority populations who can be disproportionately impacted by crises and disasters, often due to a lack of clear and accessible information.^3–5^ In an attempt to encourage more effective participation, solutions often centre on ensuring that translated information about the emergency is available and meets appropriate readability levels.^6^ These strategies are important but insufficient and often fraught with cultural, timeliness and mistranslation issues.^5 7^ For instance, although generic preparedness information may remain relevant for an extended period and can be made available in various languages ahead of disasters, it is not always possible to translate ‘live’ emergency information and machine or artificial intelligence (AI)-driven communication, although useful, is not without problems.^8^ Another significant challenge in effectively engaging ethnic minority communities in emergencies is the impracticality of governments delivering comprehensive information in all languages during crises characterised by linguistic and cultural diversity and rapidly evolving communication demands. Consequently, tailored and pragmatic communication and engagement strategies that leverage community members’ and organisations’ expertise and insights are essential for the behaviour change required to implement plans.

During a public health crisis, meaningful engagement with community members and organisations representing varied ethnic, linguistic and religious backgrounds is vital to ensuring culturally informed and effective interventions. Their support for community outreach activities through their involvement in decision-making, planning, design, governance and service delivery is also essential.^9 10^ Specific community members who are considered part of the solution for supporting community prevention efforts during pandemics and health crises include community leaders, faith leaders, popular opinion leaders, bilingual case workers or other front-line staff in community-based organisations (CBOs), as well as informal volunteers within the emergency response sector (CBOs are non-profit organisations, either public or private, that encompass faith-based groups, community coalitions, community development corporations and non-governmental social service agencies).^11 12^ These actors are often described as trusted sources of information and support for community members.^13–16^ They are also well-placed to identify solutions, given their understanding of the knowledge and rumours circulating in their communities.^11^ They are well-positioned to collaborate with others to develop collective responses.

While the literature notes that these actors have been involved to varying degrees in the COVID-19 pandemic response and during other emergencies, the emphasis has been on their contributions to response efforts.^17–22^ In high income settings, the level of trust placed in these actors, and how that trust is developed and maintained across different types of crises and disasters, remains poorly understood. Trust has historically been recognised as fundamental to the effective implementation of public health measures,^23^ as it ‘allows a person with less knowledge, power or ability to process complex information, to rely on another individual or institution to make decisions aligned with their well-being’ (^24^,p.266). Accordingly, examining both the level of trust afforded to these actors in emergency contexts and the factors that influence it is vital, though evidence on these dimensions remains limited.

Much of the literature around community engagement strategies and the roles of CBOs has come from events such as outbreaks of Ebola, Zika, MPox and the ongoing burden of malaria and yellow fever in low-resource settings.^25 26^ In these settings, engagement with CBOs may align with land ownership, family ties with village authorities or economic status.^27^ However, in high-income settings, we do not necessarily have the same level of understanding regarding the interaction of ethnic minority community members with community leaders and CBOs. Nor do we fully understand the communities’ perceptions of communication efforts by these volunteers and CBOs during emergencies, whether these efforts build public trust or translate into action. Furthermore, as much of the research in this space is qualitative, with limited sample sizes, the objectivity and generalisability of the data are limited.^28^ Generating quantitative evidence on trust in leaders and community organisations has the potential to enhance evidence-to-policy efforts by providing a more robust empirical foundation and by illuminating areas where further research is needed.^29^ This study aimed to address the research gaps previously outlined by exploring the levels of and perceptions of trust by multicultural community members towards community leaders and CBOs and the interaction and influence of these actors during crises in Australia.

## Methods

### Study design and participants

This study employed a cross-sectional survey design. Participants were eligible if they were aged 18 years or older and identified as belonging to one of the following community groups: Chinese, Arabic, Ezidi, Nepalese, Bangladeshi or Pasifika. These groups were selected through iterative discussions with the study team, which included members working in multicultural health, communications and refugee health and in consultation with the project steering committee comprising nine lay members from ethnically diverse backgrounds. The aim was to identify community groups that varied in terms of (1) population size (2) degree of establishment within Australia (ie, whether they had dedicated community organisations/associations) or whether they were considered a community that was ‘new and emerging’ and (3) whether we had established community links. In Australia, Culturally and Linguistically Diverse is the preferred term for non-indigenous ethnic minorities. It comprises first-generation, second-generation and third-generation migrants and refugees with cultural and/or language connections to their country or ancestry of origin.^30^

Recruitment was restricted to the three largest states in Australia: New South Wales (NSW), Victoria (VIC) and Queensland (QLD), as this study was part of a larger, funded study. The sample size for the cross-sectional survey was 385 to estimate a prevalence of 50% with an accuracy of ±5% with 95% confidence. Proportional quota sampling was used to ensure respondents were demographically representative of the selected community groups, with quotas set by language/community group and state. For each focus community, we identified and recruited peer researchers who spoke the same language or self-identified as members of that community. We recruited and trained one or two peer researchers from each focus community and state. In addition, information about the study and the survey link was sent to various CBOs, including those dedicated to the community group or more generic multicultural organisations. The participant information sheet, survey link and survey questions were all translated into the target languages (for Chinese, this included Traditional and Simplified). For the Pasifika community members, the survey was distributed in English, the common language for communication. Potential participants were offered multiple options to participate: completing the survey online, completing a paper-based survey (available in English or translated), or having a bilingual peer researcher enter their responses into the survey platform on their behalf. No incentives were provided for survey participation. After reading the participant information, consent was implied if the person completed the survey and submitted it via the QOR website. No personal identifiers were collected. Duplicate entries were prevented by recording each respondent’s unique IP address. The study was conducted and reported in accordance with the Strengthening the Reporting of Observational Studies in Epidemiology (STROBE) guidelines for cross-sectional studies (online supplemental file 1).^31^

### Survey design

The survey questions were developed using several validated tools, including the Sense of Community Scale,^32^ the Social Provisions Scale,^33^ the Gatekeeper Performance Scale and the Perceived Value tool.^34^ In addition, several other questions were added to explore exposure to community-led activities during the COVID-19 pandemic. The survey included questions focused on perceptions towards support received from ‘their community’ before COVID and during COVID, participation in community groups/associations, sense of belonging, frequency of and perceptions towards engaging with CBOs and community/religious leaders during the COVID pandemic and sense of trust and comfort towards interacting with these organisations/leaders during an emergency. In addition to focusing on COVID, we repeated the questions and asked participants to reflect on their experiences with natural disasters and heat waves. Participant exposure to an event was captured, along with their exposure to receiving support (information to help prepare, warnings, information/practical help post-event) from a community organisation or leader and perceptions of the effectiveness/usefulness of the CBOs/leaders. To support a common understanding of ‘community’, we highlighted that this could be their town, suburb or local government area. We also used the word neighbourhood to denote people within their localised area. Sociodemographic characteristics collected included gender, age, education level, marital status, languages spoken, religion, time spent in the current neighbourhood and birthplace/ethnic origin. Surveys were available in English or translated, using translators with National Accreditation Authority for Translators and Interpreters accreditation where possible. Surveys were hosted on the Qualtrics web-based survey platform.

### Data analysis

Descriptive analyses were computed for the survey responses and demographic information. Trust was then dichotomised into very low-to-low trust (scores 1–3 on a 7-point Likert scale) and moderate-to-very high trust (scores 4–7). Missing data were assessed to be randomly distributed and the missing values were imputed using multiple imputations. Multiple binomial logistic regression models were used to determine the factors associated with moderate-to-very high trust in (1) CBOs and (2) community leaders. Education, age group, gender, country of birth, trust in several sources, interactions with CBOs, interactions with community leaders and communication preferences. The model also controlled for community group and state. Estimates were pooled across multiple imputations using Rubin’s rules. No multicollinearity among variables was identified. P values of <0.05 were considered statistically significant. Data analyses were conducted using SPSS V.29.0 and R Studio. Free-text fields were manually analysed and grouped into common themes.

### Patient and public involvement

Public involvement in this study was sought across all phases. Study design, inclusion/exclusion criteria and data collection plans were discussed with a steering committee comprising stakeholders from emergency services, multicultural community members and CBOs. Peer researchers, as discussed above, were involved in data collection, interpretation and representation and manuscript review.

## Results

### Sample characteristics

A total of 590 respondents completed the survey, and a further 79 attempted it beyond the eligibility questions and were included in the descriptive analysis. 97 respondents were found ineligible and excluded from analysis. Most participants were current residents of NSW (372, 55.6%), followed by VIC (152, 22.7%) and QLD (145, 21.7%). A large majority (81%) were born outside of Australia (n=543), of whom 48% had arrived in the last 10 years (n=261). See table 1 for a full breakdown of participant characteristics by community group.

**Table 1:** Participant characteristics

	Arabic n (%)	Chinese n (%)	Nepalese n (%)	Bangladeshi n (%)	Ezidi n (%)	Pasifika n (%)
How do you describe your gender?						
Man/male Woman/female Non-binary/gender queer/gender-fluid I use a different term Prefer not to answer	28 (17.2) 132 (81) 0 1 (0.6) 2 (1.2)	60 (39.5) 84 (55.3) 3 (2) 1 (0.7) 4 (2.6)	31 (66) 15 (31.9) 0 0 1 (2.1)	74 (53.2) 63 (45.3) 1 (0.7) 0 1 (0.7)	18 (46.2) 18 (46.2) 1 (2.6) 0 2 (5.1)	14 (24.1) 42 (72.4) 0 1 (1.7) 1 (1.7)
Age						
18–24 25–34 35-44 45–54 55–64 65–74 75–84 85–94 ≥95 Prefer not to say	10 (6.1) 34 (20.9) 78 (47.9) 27 (16.6) 11 (6.7) 1 (0.6) 1 (0.6) 0 0 1 (0.6)	71 (46.7) 48 (31.6) 9 (5.9) 10 (6.6) 6 (3.9) 3 (2) 0 1 (0.7) 1 (0.7) 3 (2)	10 (21.3) 17 (36.2) 6 (12.8) 7 (14.9) 2 (4.3) 0 0 0 0 5 (10.6)	5 (3.6) 22 (15.8) 62 (44.6) 23 (16.5) 10 (7.2) 11 (7.9) 6 (4.3) 0 0 0	15 (38.5) 11 (28.2) 4 (10.3) 5 (12.8) 2 (5.1) 0 0 0 0 2 (5.1)	13 (22.4) 18 (31) 8 (13.8) 14 (24.1) 2 (3.4) 3 (5.2) 0 0 0 0
Were your parents born outside of Australia?						
Both parents born overseas Father only born overseas Mother only born overseas Both parents born in Australia Not stated Prefer not to say	151 (94.4) 1 (0.6) 0 3 (1.9) 0 0	134 (88.7) 2 (1.3) 0 2 (1.3) 0 11 (7.3)	39 (84.8) 1 (2.2) 1 (2.2) 0 1 (2.2) 3 (8.7)	131 (95.6) 0 0 1 (0.7) 1 (0.7) 4 (2.9)	37 (97.4) 0 0 0 0 1 (2.6)	53 (91.4) 3 (5.2) 1 (1.7) 0 0 1 (1.7)
What state do you currently reside in?						
NSW Victoria Queensland	102 (53.1) 48 (25) 42 (21.9)	108 (65.5) 36 (21.8) 21 (12.7)	29 (52.7) 25 (45.5) 1 (1.8)	71 (47.7) 40 (26.8) 38 (25.5)	35 (85.4) 6 (14.6) 0	27 (40.3) 3 (4.5) 37 (55.2)
What is the highest educational qualification you completed?						
Less than high school High school or equivalent Certificate levels I to IV Advanced diploma and diploma level Bachelor’s degree level and above Prefer not to answer	2 (1.3) 16 (10) 6 (3.8) 23 (14.4) 109 (68.1) 4 (2.5)	2 (1.3) 14 (9.3) 3 (2) 5 (3.3) 122 (80.8) 5 (3.3)	3 (6.5) 3 (6.5) 2 (4.3) 9 (19.6) 27 (58.7) 2 (4.3)	0 4 (2.9) 3 (2.2) 0 128 (93.4) 2 (1.5)	8 (21.1) 10 (26.3) 7 (18.4) 5 (13.2) 6 (15.8) 2 (5.3)	1 (1.7) 18 (31) 13 (22.4) 9 (15.5) 15 (25.9) 2 (3.4)
What is your marital status?						
Never married Married Divorced Separated Widowed	13 (8.3) 129 (82.2) 8 (5.1) 4 (2.5) 3 (1.9)	109 (75.2) 31 (21.4) 4 (2.8) 0 1 (0.7)	15 (32.6) 31 (67.4) 0 0 0	11 (8.1) 117 (86) 1 (0.7) 1 (0.7) 6 (4.4)	23 (63.9) 13 (36.1) 0 0 0	28 (48.3) 26 (44.8) 2 (3.4) 0 2 (3.4)
What is your religion?						
Islam Buddhism Hinduism Catholic Anglican (Church of England) Uniting Church Presbyterian Greek Orthodox Baptist Other No religion Other Christian denomination Ezidi	139 (86.9) 0 1 (0.6) 4 (2.5) 0 0 0 5 (3.1) 0 4 (2.5) 4 (2.5) 3 (1.9) 0	1 (0.7) 22 (14.6) 0 1 (0.7) 1 (0.7) 1 (0.7) 2 (1.3) 1 (0.7) 3 (2) 4 (2.6) 112 (74.2) 3 (2) 0	0 1 (2.2) 43 (93.5) 1 (2.2) 0 0 0 0 0 0 1 (2.2) 0 0	130 (94.9) 1 (0.7) 2 (1.5) 0 0 0 0 0 0 1 (0.7) 3 (2.2) 0 0	0 0 0 0 0 0 0 0 0 1 (2.6) 1 (2.6) 0 36 (94.7)	0 0 0 3 (5.2) 1 (1.7) 5 (8.6) 6 (10.3) 0 16 (27.6) 2 (3.4) 4 (6.9) 21 (36.2) 0
What language would you like to get information in an emergency						
English English and other language Other language	64 (33.3) 30 (15.6) 98 (51)	28 (17) 11 (6.7) 126 (76.4)	25 (45.5) 7 (12.7) 23 (41.8)	88 (59.1) 25 (16.8) 36 (24.3)	18 (43.9) 3 (7.3) 20 (48.8)	43 (64.2) 4 (6) 20 (29.9)

NSW, New South Wales.

### Trust in actors or organisations

There was a large degree of variation in levels of trust in the actors or organisations that provided information about COVID-19 across the different community groups (table 2). Participants from all groups except Pasifika reported the lowest trust in religious leaders, whereas those from Pasifika backgrounds reported the lowest trust in doctors. The most trusted information sources varied across different cultural groups. For individuals from Chinese, Arabic and Bangladeshi backgrounds, the Australian Government and doctors were the most trusted. Nepalese participants highly trusted their local council and the Australian Government. For Ezidi participants, bilingual community workers and doctors were the most trusted sources. Lastly, Pasifika participants relied primarily on religious centres and religious leaders for trustworthy information. Pasifika participants also reported increased trust in CBOs during the COVID-19 pandemic compared with before the pandemic.

**Table 2:** Participant sense of community and information sources

	Arabic n (%)	Chinese n (%)	Nepalese n (%)	Bangladeshi n (%)	Ezidi n (%)	Pasifika n (%)
Agreement (agree/strongly agree) with the following:						
I feel like a member of my community. I can rely on my community members to help me during emergencies or pandemics. Members of this community offer strong support to each other. There are organisations in my community that provide support. There are leaders in my community who support us. Religious leaders in my community provide support. I feel a sense of belonging to this community.	160 (85.5) 157 (82.7) 148 (77.8) 139 (74.8) 101 (53.7) 125 (66.1) 152 (80)	146 (89) 128 (78.1) 126 (76.4) 133 (81.6) 100 (61.4) 80 (49.1) 123 (75.5)	50 (90.9) 44 (80) 36 (78.2) 38 (70.4) 36 (68) 27 (52) 40 (75.5)	140 (94) 134 (89.9) 124 (83.2) 101 (68.3) 92 (61.7) 101 (68.3) 132 (88.6)	40 (97.5) 39 (97.5) 39 (97.5) 33 (86.8) 31 (77.5) 29 (74.4) 38 (100)	60 (89.6) 60 (89.5) 63 (94) 59 (88) 61 (91) 60 (89.6) 60 (89.6)
Within your suburb, city or town, how many close relatives do you have						
None 1–5 6–10 Over 10	77 (48.7) 49 (31) 12 (7.6) 20 (12.7)	66 (44.3) 58 (38.9) 17 (11.4) 8 (5.4)	6 (15) 22 (55) 7 (17.5) 5 (12.5)	68 (51.1) 48 (36.1) 11 (8.3) 6 (4.5)	2 (5.3) 27 (71.1) 4 (10.5) 5 (13.2)	7 (12.1) 14 (24.1) 8 (13.8) 29 (50)
Within your suburb, city or town, how many close friends do you have						
None 1–5 6–10 Over 10	26 (17.9) 64 (44.1) 24 (16.6) 31 (21.4)	16 (11) 81 (55.5) 28 (19.2) 21 (14.4)	1 (3.1) 19 (59.4) 4 (12.5) 8 (25)	12 (8.9) 72 (53.3) 21 (15.6) 30 (22.2)	0 7 (25.9) 13 (48.1) 7 (25.9)	2 (3.6) 15 (26.8) 11 (19.6) 28 (50)
Within your suburb, city or town, how many people in your neighbourhood that you know						
None 1–5 6–10 Over 10	20 (12.8) 68 (43.6) 18 (11.5) 50 (32.1)	18 (11.9) 70 (46.4) 29 (19.2) 34 (22.5)	9 (23.1) 19 (48.7) 3 (7.7) 8 (20.5)	13 (9.6) 67 (49.3) 22 (16.2) 34 (25)	0 10 (27) 11 (29.7) 16 (43.2)	6 (10.3) 19 (32.8) 7 (12.1) 26 (44.8)
Within your suburb, city or town, how many people in your neighbourhood you know well enough to ask for a favour (picking up the mail, watering plants, etc)						
None 1–5 6–10 Over 10	45 (28.8) 74 (47.4) 23 (14.7) 14 (9)	36 (24) 83 (55.3) 21 (14) 10 (6.7)	10 (25.6) 20 (51.3) 6 (15.4) 3 (7.7)	33 (24.3) 73 (53.7) 11 (8.1) 19 (14)	1 (2.6) 9 (23.7) 14 (36.8) 14 (36.8)	13 (22.8) 20 (35.1) 7 (12.3) 17 (29.8)
	Mean (SD, N)	Mean (SD, N)	Mean (SD, N)	Mean (SD, N)	Mean (SD, N)	Mean (SD, N)
Trust in the following sources to provide relevant information about COVID-19 (1=low trust, 7=high trust)						
Community group linked to your culture or ethnicity Your local council Religious centres such as a church, mosques, temples, synagogues and others The Australian Government A religious leader A doctor who speaks your language or is from your community A community leader A community worker who speaks your language or is from your cultural background	4.04 (1.56,188) 4.33 (1.70,186) 4.18 (1.74,183) 4.87 (1.70,191) 3.73 (1.87,173) 4.78 (1.69,186) 3.55 (1.70,173) 3.88 (1.79,178)	4.56 (1.43,158) 4.08 (1.58,154) 3.10 (1.70,130) 4.66 (1.59,152) 2.75 (1.64,117) 4.68 (1.64,151) 3.42 (1.61,132) 4.26 (1.70,141)	4.89 (3.18,52) 6.34 (3.33,53) 4.64 (3.63,50) 5.81 (2.55,53) 4.08 (3.22,52) 5.40 (3.70,52) 5.43 (3.79,53) 5.04 (3.35,53)	4.56 (1.60,143) 4.70 (1.62,140) 4.15 (1.84,143) 5.61 (1.46,147) 3.91 (1.96,138) 5.49 (1.53,143) 4.04 (1.88,143) 4.32 (1.77,139)	4.85 (1.44,39) 4.50 (1.39,38) 3.94 (1.30,35) 5.14 (1.27,37) 4.11 (1.54,37) 5.45 (1.69,38) 5.14 (1.38,35) 5.58 (1.32,36)	4.33 (1.71,66) 4.00 (1.95,66) 4.65 (1.95,66) 4.05 (2.13,66) 4.61 (1.97,66) 3.86 (2.22,65) 4.32 (1.94,66) 4.52 (1.91,65)

### Community-based organisations

Across community groups, participants most commonly reported neutral levels of satisfaction with CBO communication during the pandemic, ranging from 29.4% to 41.3% (table 3). Attendance at CBO-organised events, both virtual and in person, declined during the pandemic compared with pre-pandemic levels in all community groups (online supplemental figure 1). Patterns of contact from CBOs remained largely stable, although Nepalese and Ezidi participants reported an increase in frequent communication during the pandemic (online supplemental figure 2). Similarly, the likelihood of seeking help or support from CBOs showed little overall change across most groups. However, Ezidi and Nepalese participants indicated a greater likelihood of doing so during the pandemic than before (online supplemental figure 3).

**Table 3:** Organisations’ support of community during COVID-19 and future emergencies

	Arabic n (%)	Chinese n (%)	Nepalese n (%)	Bangladeshi n (%)	Ezidi n (%)	Pasifika n (%)
During the COVID-19 pandemic, how satisfied were you with how community organisations communicated to your community?						
Very satisfied Satisfied Neutral Dissatisfied Very dissatisfied Did not experience	17 (9.5) 56 (31.3) 67 (37.4) 18 (10.1) 5 (2.8) 16 (8.9)	11 (6.9) 52 (32.5) 66 (41.3) 3 (1.9) 3 (1.9) 25 (15.6)	6 (11.8) 22 (43.1) 15 (29.4) 1 (2) 2 (3.9) 5 (9.8)	13 (9.1) 46 (32.2) 52 (36.4) 4 (2.8) 5 (3.5) 23 (16.1)	5 (12.5) 9 (22.5) 22 (55) 4 (10) 0 0	9 (14.3) 19 (30.2) 26 (41.3) 4 (6.3) 3 (4.8) 2 (3.2)
In future health emergencies, what information do you think community organisations should provide						
Information about recommended safety measures Updates on the pandemic situation (eg, case numbers, vaccination rates) Changes in government regulations or policies Information on testing centres and vaccination sites Updates on local community resources and support services Mental health and well-being resources Resources to help with navigating Australian government websites or services	123 (64.1) 98 (51) 80 (41.7) 107 (55.7) 102 (53.1) 101 (52.6) 86 (44.8)	118 (71.5) 108 (65.5) 114 (69.1) 117 (70.9) 103 (62.4) 96 (58.2) 72 (43.6)	38 (69.1) 3 (60) 30 (54.5) 36 (65.5) 37 (67.3) 41 (74.5) 36 (65.5)	112 (75.2) 92 (61.7) 99 (66.4) 107 (71.8) 103 (69.1) 106 (71.1) 90 (60.4)	28 (68.3) 25 (61) 22 (53.7) 18 (43.9) 13 (31.7) 15 (36.6) 18 (43.9)	40 (59.7) 38 (56.7) 47 (70.1) 34 (50.7) 47 (70.1) 49 (73.1) 40 (59.7)
	Mean (SD, N)	Mean (SD, N)	Mean (SD, N)	Mean (SD, N)	Mean (SD, N)	Mean (SD, N)
Trust in community organisations before the COVID-19 pandemic (1=low trust, 7=high trust)	3.70 (1.69,179)	4.02 (1.51,160)	3.90 (1.58,51)	4.21 (1.66,142)	4.43 (1.36,40)	4.59 (1.68,63)
Trust in community organisations during the COVID-19 pandemic (1=low trust, 7=high trust)	3.78 (1.71,176)	4.03 (1.50,159)	4.16 (1.74,51)	4.31 (1.79,143)	5.08 (1.57,38)	4.48 (1.74,63)

### Determinants of trust in community organisations

A binary logistic regression model (see table 4) identified that the variables associated with moderate-to-very high trust in CBOs during COVID-19 were: identifying as being a part of the Nepalese community (OR=3.242); having a higher likelihood of asking for help or information from a CBO during COVID-19 (OR=2.276); arriving in Australia ≥20 years ago (OR=2.897); having moderate-to-very high trust in a community group linked to the person’s culture or ethnicity (OR=2.163), engaging with local council (OR=2.012), or engaging with a community worker who spoke the same language or was from the same community (OR=2.147). In contrast, being very dissatisfied with (OR=0.152) or not experiencing (OR=0.153) communication from CBOs to the participants’ community during COVID-19 was inversely associated. All other variables showed no association.

**Table 4:** Predictors of moderate-to-high trust in community-based organisations (CBOs) and community leaders during COVID-19 (n=545)

	CBOs				Community leaders			
Factors	OR	95% CI		P value	OR	95% CI		P value
State								
NSW* VIC QLD	0.789 0.791	0.403 0.390	1.546 1.607	0.491 0.518	1.496 1.108	0.736 0.522	3.043 2.352	0.267 0.790
Community								
Arabic* Chinese Nepalese Bangladeshi Ezidi Pasifika	2.088 3.242 1.188 0.632 1.051	0.814 1.035 0.526 0.143 0.344	5.183 10.150 2.681 2.791 3.210	0.113 0.044 0.678 0.546 0.931	1.263 2.182 1.824 0.749 1.338	0.519 0.642 0.782 0.146 0.303	3.072 7.414 4.257 3.823 3.655	0.606 0.212 0.165 0.728 0.935
I can rely on my community members to help me during emergencies or pandemics								
Disagree-to-strongly disagree* Agree-to-strongly agree	1.277	0.864	1.888	0.220	1.338	0.881	2.034	0.173
Frequency of going to events organised by CBOs before COVID	1.344	0.991	1.823	0.06				
Frequency of going to events organised by CBOs during COVID	1.317	0.924	1.876	0.127				
Frequency of interaction with community leaders before COVID					0.589	0.367	0.947	0.029
Frequency of interaction with community leaders during COVID					2.371	1.408	3.992	0.001
Frequency of contact by CBOs before COVID	0.944	0.632	1.411	0.781				
Frequency of contact by CBOs during COVID	1.266	0.856	1.872	0.238				
Comfortability in talking to community leaders before COVID					1.561	0.944	2.579	0.083
Comfortability in talking to community leaders during COVID					1.453	0.870	2.427	0.154
Likelihood of asking for help or information from a CBO/community leader before COVID	0.769	0.526	1.124	0.176	1.223	0.764	1.959	0.402
Likelihood of asking for help or information from a CBO/community leader during COVID	2.276	1.569	3.299	**<**0.001	1.733	1.093	2.747	0.020
Satisfaction with how CBOs communicated to your community during COVID-19								
Very satisfied* Satisfied Neutral Dissatisfied Very dissatisfied Did not experience	0.608 0.373 0.380 0.152 0.153	0.168 0.107 0.078 0.024 0.036	2.206 1.293 1.851 0.967 0.648	0.450 0.120 0.231 0.047 0.011				
Gender								
Man/male* Woman/female	0.917	0.524	1.605	0.762	0.618	0.338	1.128	0.117
Country of birth								
Australia* Outside of Australia	2.950	0.760	11.447	0.119	1.904	0.822	4.414	0.134
Arrival year in Australia								
Last 5 years* Between 5 and 10 years Between 10 and 20 years 20+ years	1.820 1.142 2.897	0.807 0.502 1.102	4.104 2.598 7.616	0.150 0.751 0.**032**	1.904 1.515 2.283	0.822 0.630 0.791	4.414 3.643 6.590	0.134 0.354 0.128
Moderate to very high trust in sources (*very low to low trust)								
Community group linked to your culture or ethnicity Local council Religious centres The Australian Government A religious leader A doctor who speaks your language or is from your community A community leader A community worker who speaks your language or is from your community	2.163 2.012 1.331 1.411 1.044 0.744 1.164 2.147	1.113 1.077 0.635 0.728 0.454 0.375 0.568 1.134	4.205 3.760 2.789 2.736 2.399 1.476 2.384 4.068	0.024 0.029 0.450 0.308 0.919 0.398 0.678 0.020	2.658 2.462 1.628 0.658 – 1.731 – 1.186	1.340 1.234 0.854 0.302 – 0.839 – 0.620	5.271 4.911 3.103 1.431 – 3.571 – 2.268	0.005 0.011 0.140 0.292 – 0.138 – 0.606
Age								
18–24* 25–34 35–44 45–54 55–64 65–74 75+	0.977 1.387 1.146 0.427 0.590 1.196	0.163 0.323 0.295 0.129 0.214 0.530	5.844 5.964 4.442 1.412 1.629 2.701	0.980 0.660 0.844 0.164 0.309 0.667	1.875 8.437 1.650 0.784 0.762 0.706	0.253 1.556 0.362 0.207 0.242 0.286	13.879 45.746 7.522 2.969 2.396 1.743	0.539 0.014 0.518 0.721 0.642 0.451
Language wanted for future emergencies								
English* English and other language Other language	0.842 0.844	0.388 0.453	1.829 1.574	0.664 0.595	1.196 1.200	0.513 0.630	2.789 2.283	0.679 0.579
Education								
Less than high school* High school or equivalent Certificate levels I to IV Advanced diploma and diploma level Bachelor’s degree level and above	0.363 0.287 0.275 0.340	0.040 0.029 0.031 0.042	3.316 2.810 2.471 2.735	0.371 0.285 0.250 0.311	0.079 0.164 0.253 0.107	0.005 0.010 0.015 0.008	1.256 2.768 4.180 1.469	0.074 0.211 0.338 0.096

* Reference variable.

NSW, New South Wales; OR, log OR controlling for other variables in the model; p, probability value (statistically significant <0.05); QOL, Queensland; VIC, Victoria.

### Community leaders

Participants reported mostly ‘neutral’ satisfaction (from 35.6% to 63.2% across community groups) towards communication from community leaders during COVID-19 (table 5). Each community group, except for Pasifika participants (who remained consistently positive), reported a very slight increase in trust towards community leaders during the COVID-19 pandemic compared with before the pandemic. Responses related to information received from community leaders during the COVID-19 pandemic varied across community groups, with health guidelines and safety measures, updates on local COVID-19 cases and testing facilities and Government regulations among the most frequently reported. Community support resources (food banks, mental health services, etc) and economic support and financial assistance programmes were among the least reported support mechanisms accessed.

**Table 5:** Community leader support of community during COVID-19 and future emergencies

	Arabic n (%)	Chinese n (%)	Nepalese n (%)	Bangladeshi n (%)	Ezidi n (%)	Pasifika n (%)
During the COVID-19 pandemic, how satisfied were you with how community leaders communicated to your community?						
Very satisfied Satisfied Neutral Dissatisfied Very dissatisfied Did not experience	12 (7.5) 51 (31.7) 70 (43.5) 10 (6.2) 6 (3.7) 12 (7.5)	11 (7.3) 39 (26) 59 (39.3) 7 (4.7) 1 (0.7) 33 (22)	4 (8.9) 19 (42.2) 16 (35.6) 2 (4.4) 1 (2.2) 3 (6.7)	15 (10.8) 36 (25.9) 55 (39.6) 8 (5.8) 3 (2.2) 22 (15.8)	4 (10.5) 10 (26.3) 24 (63.2) 0 0 0	5 (8.6) 19 (32.8) 27 (46.6) 6 (10.3) 0 1 (1.7)
What types of information did you receive from community leaders during the COVID-19 pandemic? Please select all that apply from the following options:						
Health guidelines and safety measures Updates on local COVID-19 cases and testing facilities Government regulations and restrictions Community support resources (food banks, mental health services, etc) Vaccination information and distribution updates Educational resources for remote learning Economic support and financial assistance programmes	90 (46.9) 75 (39.1) 68 (35.4) 44 (22.9) 69 (35.9) 55 (28.5) 43 (22.4)	80 (48.5) 80 (48.5) 68 (41.2) 64 (38.8) 66 (40) 38 (23) 43 (26.1)	26 (47.3) 28 (50.9) 23 (41.8) 25 (45.5) 29 (52.7) 14 (25.5) 18 (32.7)	83 (55.7) 74 (49.7) 66 (44.3) 48 (32.2) 79 (53) 32 (21.5) 30 (20.1)	31 (75.6) 26 (63.4) 20 (48.8) 9 (22) 16 (39) 19 (46.3) 7 (17.1)	35 (52.2) 29 (43.3) 38 (56.7) 26 (38.8) 32 (47.8) 8 (11.9) 16 (23.9)
	Mean (SD, N)	Mean (SD, N)	Mean (SD, N)	Mean (SD, N)	Mean (SD, N)	Mean (SD, N)
Trust in community leaders before the COVID-19 pandemic (1=low trust, 7=high trust)	3.80 (1.64, 160)	4.01 (1.48, 149)	3.68 (1.62, 47)	4.23 (1.76, 138)	4.42 (1.48, 38)	4.34 (1.72, 58)
Trust in community leaders during the COVID-19 pandemic (1=low trust, 7=high trust)	3.87 (1.77, 160)	4.02 (1.46, 151)	4.13 (1.80, 47)	4.31 (1.79, 138)	5.05 (1.55, 37)	4.30 (1.61, 57)

The frequency of interactions with community leaders increased only among participants from Pasifika backgrounds; for all other groups, interactions were generally reported as rare or absent both before and during the COVID-19 pandemic (online supplemental figure 4). Levels of comfort in speaking with community leaders (online supplemental figure 5) and the likelihood of seeking help or information from them (online supplemental figure 6) showed minimal change across most groups during the pandemic. However, participants from Ezidi and Nepalese backgrounds reported an increased likelihood of seeking help or information

### Determinants of trust in community leaders

A binary logistic regression model (see table 4) identified that the variables associated with moderate-to-very high trust in community leaders during COVID-19 were: higher levels of interaction with community leaders during COVID-19 (OR=2.371); likelihood of asking for help or information from a community leader during COVID-19 (OR=1.733); moderate-to-very high trust in a community group linked to culture or ethnicity (OR=2.658), interaction with local council (OR=1.628); and being aged between 35 and 44 (OR=8.437). In contrast, the frequency of interaction with community leaders before COVID-19 (OR=0.589) was inversely associated. All other variables showed no association.

## Discussion

Evidence suggests that community leaders and other community helpers can strengthen social connections within communities and act as a key conduit between official government services and community members.^35 36^ These dynamics have historically been understood through a social capital lens, where trust is shaped by social networks, prior interactions and ongoing engagement within communities.^37^ Within this framework and what we identified in our work, we found that trust in community leaders and organisations is not assumed but develops through repeated contact, familiarity and perceived reciprocity. Despite its wide utility, the social capital theory has notable limitations, including conceptual ambiguity, measurement difficulties and concerns about causality. Furthermore, social capital can have unintended negative effects, such as reinforcing social exclusion or privileging already well-connected groups, which is particularly relevant when considering diverse and marginalised communities.^38^

Our findings indicate that trust in community leaders, CBOs and government agencies is closely linked to individuals’ likelihood of seeking help or information and to the frequency of interaction during a health crisis. Rather than being inherent to specific actors, trust appears to emerge through engagement, suggesting that individuals who are already connected to community structures are more likely to view these actors as credible and trustworthy. This aligns with diffusion of innovations theory, where trusted individuals and organisations act as key channels through which information and behaviours spread within social networks.^39^ This finding also reflects similar results to research in civic engagement in Australia and social capital in Norway, where group membership, specifically the number of group memberships per person and to a lesser extent intensity of involvement, are both important predictors of civic engagement.^40 41^ This is an important finding and a reminder to governments that they cannot assume that multicultural community members align with community organisations or leaders simply because an emergency is occurring. It also reinforces the need for CBOs to invest in outreach and engagement activities both before and during emergencies, including efforts to better understand the priorities and needs of the communities they serve. Such engagement is central to building the relational foundations required for effective crisis communication.

Perhaps unsurprisingly, we found that arrival in Australia ≥20 years ago was a significant predictor of higher trust in CBOs, which may reflect the accumulation of social capital over time. Longer-term residents may have had more opportunities to establish networks, engage with community organisations and develop familiarity with local systems. This finding highlights the importance of targeted engagement strategies for newer arrivals, who may have fewer established connections and therefore lower baseline trust. To ensure meaningful and relevant responses during emergencies, the WHO advocates for early and sustained community engagement, including fostering partnerships that enhance trust and support public health measures.^42^

Among participants, there were varying levels of trust in different information sources, with most reporting the highest trust in the Australian Government and medical professionals and the least trust in religious leaders. Only among Pasifika participants did we observe a different pattern, with lower trust in doctors and higher trust in religious centres and leaders. Notably, trust in community groups and local councils was a significant predictor of trust in both CBOs and community leaders. From a social capital perspective, this suggests that trust may transfer across interconnected networks, where confidence in one local actor reinforces trust in others within the same ecosystem. We acknowledge that our study was conducted in 2023 and not at the height of the COVID-19 pandemic, which may have influenced responses. However, these findings align with previous research and broader evidence indicating consistently high levels of trust in healthcare providers across settings.^35 36^ These findings also align with a recent systematic review, which examined 41 studies on previous pandemics, epidemics and global outbreaks in the 21st century. While the authors identified inconsistencies in public trust towards the government and the media across multiple countries over time, trust in healthcare providers was generally reported to be high, with a few exceptions.^43^

There is limited COVID-19-specific research examining patterns of trust and the factors shaping community confidence in CBOs and community leaders. Existing qualitative and quantitative studies on information preferences during the pandemic have largely centred on the roles of government agencies, healthcare providers, social media and family or friends. Although many accounts describe CBOs as having played a critical role in supporting the COVID-19 response, there is comparatively little empirical evidence to substantiate these claims. For example, Dada *et al* highlighted the role of community partners, including Black faith-based leaders, in the COVID-19 vaccine programme as part of a review of strategies to promote COVID-19 vaccine equity. Still, there are no data to support the statements being made.^44^ Similarly, in a paper exploring the roles of religious communities during the early stages of COVID, the authors highlighted that a possible solution to vaccine hesitancy was to involve religious authorities in health awareness and vaccine promotion. Again, there was no evidence cited about the level of impact of these leaders (even in events before COVID) that supports the strong statements about their role in promoting vaccine uptake and reducing misinformation.^45^ In bringing attention to this issue, the point is not to criticise the work that has been done, but rather to ensure we do not make assumptions about emergency planning or implementation that do not align with community needs. In our study, community leaders or faith leaders may not play as critical a role as other actors or organisations in some ethnic minority communities. For example, the Chinese in particular do not necessarily see themselves as having ‘community leaders’ at all. However, for other groups, it seems clear that faith leaders are recognised as instrumental to communication efforts during an emergency, especially for those aged 35–44 who had the strongest trust in community leaders. Understanding these nuances across different cultural and linguistic groups is critical. Otherwise, governments, health organisations, or emergency management organisations may rely on community actors who may not have the hoped-for significant influence in their communities. It also means that in the period between emergencies, it’s important we move beyond assumptions about which actors diverse communities trust, and endeavour to bring to light perceptions, lived experiences, and other reliable data to support decisions about which actors might effectively assist during crises. Future studies should explore this finding in greater depth and across a broader range of population groups to confirm it across cultures.

As Wong *et al* suggest, CBOs and their staff are ‘well-equipped with the cultural acuity, language capacity and familiarity with local norms to improve structural gaps’.^46^ Beyond positive reports about CBOs, findings from this study also show that trust in a community worker who speaks the same language or is from the same community group predicts higher trust in CBOs. They lead in providing culturally responsive, in-language resources, often filling a void left by governments. Their hallmark is often the flexibility they can take to support community outreach. However, it is important to recognise that these organisations may face the same issues related to community pushback and hesitancy that governments and health officials experience. To reduce these issues during future health emergencies, CBOs must establish themselves as credible response partners during non-emergency periods. One suggestion to assist this process is to conduct a Data Walk.^47^ In this event, CBOs can hear from their communities about the challenges experienced during COVID-19 to elicit feedback on how the CBO supported efforts and to brainstorm solutions to enhance communication and engagement during future emergencies. Having discussion prompts and parameters for the conversations can facilitate this process. Not only will an event like this identify the strategies that the CBOs need to consider in the future but it will also help build authentic relationships and trust with the community as they will see these activities as promoting bidirectional communication. To support these organisations in preparing for future emergencies, governments must invest in soft infrastructure during non-emergency conditions to ensure CBO’s capacity to support emergencies/crises.^48^

Early in the pandemic, Gilmore *et al* reviewed community engagement strategies used for infectious disease prevention during epidemics and highlighted three key issues in high-resource settings: weak pre-existing community engagement structures, a limited definition of leadership roles and inadequate training and resources for community leaders and helpers.^49^ Picking up on the issue of definition, in this study, we used ‘community leader’ to explore the interaction between community members and those postulated to play a role in the distribution of information and public health messages. This term was adopted in accordance with the nomenclature used in Australia’s pandemic response plan and international plans, including those of the WHO. A ‘community leader’ is traditionally understood as someone who is seen as the ‘model representative’ of any given community. However, based on conversations about our findings with stakeholders and peer researchers, it has been suggested that in the scope of the response to COVID, the use of the term ‘community leader’ was too narrow and may not have sufficiently allowed for the capture of all the actors within a community who played a role in supporting their communities. This issue has probably influenced the data collected regarding the proportion of community members who identified as engaging with a ‘community leader’. We likely recorded a low engagement rate because of this narrow term. Subsequent conversations have highlighted that some communities may not necessarily have identifiable leaders within their communities (which may be defined by geography) or that others in the community may have been playing a role. Based on these conversations, our team has decided to use the term ‘community connector’ in future discussions and research to capture the broader scope of actors who could be assisting during a health emergency. It is important to capture the most relevant term, as it has implications for future activities during pre-emergencies and emergencies. Focusing first on preemergency periods, our findings suggest that changes in the language in national pandemic plans (or strategies for other emergencies) may be needed to ensure that the focus is not just on the potential role of so-called community ‘leaders’ but instead highlights the need for governments to engage with, train, fund and support a broader group of actors. Future research should be conducted to validate these implications and inform future changes.

Our study has several other limitations. Participant recruitment was conducted through peer researchers and CBOs, which may have limited the survey’s geographic scope to those within known networks. To reduce non-representative sample bias, the survey was available in the language to support a wider range of participants completing the study. It was available via hard copy and online. However, we acknowledge that the survey results are likely not fully representative of the six communities we focused on, as we were unable to achieve proportional quota sampling for each group. Because of this, some findings yielded wide CIs, which may limit generalisability. Future research should be conducted with more participants to provide stronger evidence of the relationship between trust and actor. Additionally, the findings may not be generalisable to other ethnic minority communities in Australia or elsewhere. In the framing of ‘community’, there was a focus on geography and on attempts to represent shared values, customs and assets. However, defining community was more challenging in the larger cities and urban centres due to a lack of cohesion, high density, informal settlement conditions and high mobility.^50^ For example, Arabic communities range in country of origin, cultural beliefs, religions, etc.

## Conclusions

Community helpers and CBOs are essential in supporting communication efforts during pandemics and other health emergencies. However, under non-emergency conditions, they must continue building relationships with relevant community members. Importantly, governments must not make assumptions about ethnic minority communities’ needs and capacities regarding their level of engagement with CBOs and community connectors during emergencies. Governments must consider the diversity between and within different ethnic minority communities. Consequently, this requires tailored, multisectoral approaches that draw on formal and informal communication strategies, including social media, community radio/newspaper and bilingual health and community workers.

## Supplementary material

10.1136/bmjgh-2025-019445 online supplemental figure 1

10.1136/bmjgh-2025-019445 online supplemental figure 2

10.1136/bmjgh-2025-019445 online supplemental figure 3

10.1136/bmjgh-2025-019445 online supplemental figure 4

10.1136/bmjgh-2025-019445 online supplemental figure 5

10.1136/bmjgh-2025-019445 online supplemental figure 6

10.1136/bmjgh-2025-019445 online supplemental file 1

## Data Availability

Data are available on reasonable request.
